# Sociocultural and structural determinants of healthcare-seeking of people affected by leprosy in Sierra Leone’s Western area: a qualitative study

**DOI:** 10.1186/s41182-025-00772-y

**Published:** 2025-07-17

**Authors:** Alexandra Asboeck, Lansana Hassim Kallon, Fabian Schlumberger, Matthew Willis, Anil Fastenau, Anja Krumeich

**Affiliations:** 1 Department of Health, Ethics and Society, Faculty of Health, Medicine and Life Sciences, Maastricht University, Maastricht, The Netherlands; 2 German Leprosy and Tuberculosis Relief Association (GLRA-MRC/DAHW), Mano River Cluster, Freetown, Sierra Leone; 3 Marie Adelaide Leprosy Center (MALC), Karachi, Pakistan; 4 Department of Global Health, Institute of Public Health and Nursing Research, University of Bremen, Bremen, Germany; 5 German Leprosy and Tuberculosis Relief Association (DAHW), Würzburg, Germany

**Keywords:** Leprosy, Healthcare-seeking behavior, Diagnostic delays, Community-based healthcare, Traditional healing, Community health workers

## Abstract

**Background:**

Leprosy, a neglected tropical disease, remains a major global health concern. If left untreated, it can lead to permanent disabilities and severe social stigma, profoundly impacting the well-being of those affected and their families. Leprosy is a public health concern in Sierra Leone, affecting hundreds of people each year. A significant number of individuals are already living with disabilities at the time of diagnosis, indicating delays in detection and treatment. These delays contribute to a vicious cycle of poverty, social exclusion, and long-term health complications.

**Methods:**

This study employed a qualitative approach to explore healthcare-seeking behaviors among people affected by leprosy in Sierra Leone’s Western Area. Eighteen semi-structured interviews were conducted with individuals affected by leprosy, traditional healers, and community health workers in the Western Area of Sierra Leone in May and June 2024. An inductive thematic analysis was used to identify key patterns and factors influencing healthcare-seeking decisions. This methodology provided an in-depth understanding of the sociocultural and systemic barriers affecting early diagnosis and treatment.

**Results:**

The study found that healthcare-seeking behavior is shaped by factors at the individual, community and healthcare system levels. Individually, misconceptions about leprosy, stigmatization and financial hardship delay care. Social attitudes in communities reinforce stigmatization and isolation. At the healthcare system level, poor access, limited services, and economic motives hinder treatment. These factors, compounded by widespread poverty, create significant obstacles to timely diagnosis and care.

**Conclusions:**

The complex interplay of individual, societal, and healthcare-related factors underscores the need for a multi-faceted approach to improving leprosy care in Sierra Leone. Multi-dimensional strategies involving people affected, traditional healers, healthcare workers, community leaders and public health policymakers are needed to address the factors contributing to diagnostic delays. Strengthening community awareness, integrating traditional healing practices with biomedical medicine, and enhancing healthcare accessibility and affordability are critical to ensuring early detection and reducing the burden of leprosy in Sierra Leone.

## Background

Leprosy, caused by *Mycobacterium leprae*, is a chronic infectious disease primarily affecting the skin and peripheral nerves, which can result in chronic disfigurement and disability if diagnosis and treatment are delayed (1). The disease remains a significant public health challenge, particularly in impoverished and marginalized communities, where it imposes not only a health burden but also substantial economic, social, and psychological challenges (2–4). Leprosy still affects communities in Sierra Leone and people worldwide with over 200,000 new cases annually (4). In Sierra Leone, 182 new leprosy cases were reported in 2022. Of these, 14.84% presented with grade-2-disabilities (G2D), reflecting delayed diagnosis, and 4.4% were children under 15 years, highlighting ongoing transmission and challenges in early detection (2, 5). Early diagnosis and swift treatment with multidrug therapy provided by the World Health Organization (WHO) are critical to preventing complications and halting further transmission (4, 6–8). However, numerous barriers delay care, especially in regions with limited healthcare access. Barriers such as poverty, limited healthcare infrastructure, local beliefs, and fear of stigmatization hinder timely health-seeking behavior (9–11).

Stigmatization and social exclusion remain central challenges, both driving and resulting from delayed diagnosis and treatment (12–14). Leprosy involves biomedical, socioeconomic, psychological and spiritual dimensions that debilitate an individual progressively, unless properly treated (15, 16). Non-medical consequences of leprosy, concerning the social determinants of health include but are not limited to stigma and social exclusion, reduced access to healthcare services, lack of educational and employment opportunities, restriction of basic human rights, increased disability and early mortality (17). The way that people interpret symptoms, disease origins and treatment options significantly shaped the healthcare-seeking behavior of people affected by leprosy (10, 18, 19). Local beliefs and explanatory models seem to prompt the consultation of traditional healing practices, often delaying biomedical care (20). A study from Paraguay found local belief systems to cause late presentation of people with leprosy symptoms. Traditional treatment was often ineffective in addressing the disease, and the traditional healers (THs) rarely referred the patients to formal healthcare (21). Abubakar et al. (22) found that THs were typically sought when illness was seen as supernatural or mystical, chronic diseases and for prevention measures. Worsening of symptoms in traditional treatment was often the pivotal motivation to move to formal healthcare (21, 23). Distance to healthcare facilities and limited clinical expertise on leprosy also drive people toward traditional medicine. Studies confirm that local disease concepts and the accessibility significantly influence healthcare-seeking behavior (24, 25), while economic and spiritual factor can make traditional options preferable. Structural barriers—such as inadequate clinical expertise, insufficient rehabilitation policies, and limited support for healthcare workers, and disruptions, such as pandemics or civil unrest—obstruct effective disease management (26–29). A low level of community awareness of leprosy symptoms is another factor linked to delays in therapy (30–32). Stigmatization of people affected by leprosy, was shown to be both cause and result of late presentation to health facilities (9, 33). Research regarding leprosy and other poverty related diseases in Sierra Leone remains limited. Studies showed, that community health workers (CHWs) and the primary healthcare system play a crucial role in progressing the elimination of neglected tropical diseases, when adequately trained and involved in biomedical healthcare (34, 35). One of the most informative research has been conducted by Opala and Boillot (36), who analyzed the traditional beliefs and practices concerning leprosy of the Limba people, residing in the northern part of Sierra Leone. The study points out the importance of culturally adapted and sensitive healthcare. By identifying concepts within Limba culture that can be adapted by leprosy workers to help convey their message, treatment outcomes and disease understanding can be fostered. Another relevant study investigated the options for child healthcare in the plural health system of Sierra Leone. It found that they often combine different treatment options and emphasized the importance of social networks and collaboration within and across families to evaluate healthcare options for the sick child. This resourcefulness needs to be recognized and integrated into the healthcare systems to increase availability of healthcare services (37). Finally, there has been a study on the collaboration with THs to reduce the delay of leprosy diagnosis and improve detection of hidden leprosy patients in Sierra Leone through establishment of a local referral system (30). The findings of the study highlight, that traditional healers are critical to the diagnostic process and for minimizing delays in diagnosis. It underscored the necessity for enhanced training and collaboration between traditional healers and healthcare providers to strengthen case identification, minimize diagnostic delays and improve patient outcomes.

The review of relevant literature on healthcare-seeking behavior disclosed that personal illness theories, local beliefs, accessibility and affordability of healthcare, and stigmatization are some of the most relevant influential factors. Furthermore, the involvement of different healthcare sectors and the communities can cause difficulties in navigation of diagnosis and treatment. Addressing these challenges is vital for improving early detection, enhancing treatment outcomes, and progressing toward the elimination of leprosy. Therefore, this study examines the factors influencing healthcare-seeking behavior among people affected by leprosys in Sierra Leone. A deeper understanding of these factors is essential for addressing diagnostic delays, preventing disabilities, and tailoring interventions to the needs of affected communities. To understand the healthcare-seeking behavior of people affected by leprosy and assess the influential factors, the following research question has been set for this study: *what are factors that influence healthcare-seeking of people affected by leprosy at individual, healthcare and societal level in Sierra Leone?*

## Methods

### Study design

A qualitative study was conducted to investigate and understand the factors that influence the healthcare-seeking behavior of people affected by leprosy in the Western Area of Sierra Leone. Semi-structured interviews were utilized to gain deeper insights into the perceptions of THs, CHWs and people affected by leprosy. The study aimed to explore the factors that ultimately contribute to diagnostic delays, and those which can be fostered for enhancing adequate healthcare-seeking behavior.

### Study site

The healthcare system in Sierra Leone operates on three levels. Peripheral health units, with the addition of CHWs, form the first level. District and referral hospitals provide outpatient and inpatient care, and diagnostic services at the second level. The tertiary level comprises of specialized hospitals, which includes seven tertiary referral hospitals (26, 38). Despite efforts to improve healthcare, only 35% of the population have access to national health services (30), and quality gaps persist due to shortages of equipment, staff, supplies and sanitation (38). With only two skilled healthcare providers per 10,000 people, far below the WHO minimum standard of 22.8 workers per 10,000 people, Sierra Leone faces a severe workforce deficit (26). Moreover, leprosy services are affected by an unequal distribution of health professionals, with 50% located in the capital Freetown. There are only 185 doctors in the country of Sierra Leone. The most recent available case notification and incidence numbers are from 2014, where there were 17 new cases of leprosy in the Western Area (26). To address workforce shortages, the national CHW program was launched in 2012, training 13,000 CHWs to bridge community and clinical healthcare through provision of basic healthcare, over the counter medication, and rapid diagnostic test. CHWs are often directly recruited from within the communities, and operate at an assigned health facility and in assigned communities (26, 28). Healthcare in Sierra Leone is not only limited in accessibility and availability, but also in affordability—with annual health expenditures at 95$ per person, 76% of which are private out-of-pocket spendings (26). Given widespread poverty, with the majority of population living below the poverty line, these costs make healthcare largely inaccessible (39, 40).

### Participant demographic and recruitment

To understand the experiences of people affected by leprosy and the management of the disease within the traditional and biomedical healthcare sector, a qualitative design was chosen. Eighteen participants were included, among them eight THs, seven people affected by leprosy and three CHWs. Semi-structured interviews were used allowing investigation of the local setting and healthcare system. Participants were selected using purposive sampling, to capture a wide range of perspectives by inclusion of diverse participants, allowing for a broad spectrum of data. Different ethnicities—Limba, Temne, Mende, Koranko and Loko—were included in every participant group of this research, to mirror the real-world situation within the communities. Participants were selected if they were over the age of 18, matched the inclusion criteria and gave their fully informed consent to be a participant of the study. THs were selected if they had prior experience in working with people affected by leprosy in terms of diagnosis, treatment and disease management. Ideally, they had experience working with members of the biomedical sector. CHWs were selected if they had experience with community health work with people affected by leprosy, to ensure a deep and thorough understanding of the situation. Ideally, they have had contact with THs in the past. Persons affected by leprosy were included if they have been diagnosed with leprosy and if they have had contact with the traditional healing system and the biomedical sector. The participant’s details are summarized in Tables 1 and 2. Descriptions of occupation were simplified to allow for the anonymity of the participants. To uncover potential urban–rural disparities in healthcare-seeking and the collaboration between biomedical and traditional medicine, participants were recruited from the Western Urban sub-district, as well as the Western Rural sub-district of the Western District, which also accommodates the capital Freetown.

**Table 1:** Participant information Persons affected by leprosy

Participant	Gender	Age	Occupation	Educational level	Years of lived experience of leprosy
Person affected by leprosy 1	Male	52	No occupation	Primary education	43
Person affected by leprosy 2	Male	75	No occupation	No formal education	41
Person affected by leprosy 3	Male	50	Community leader	Tertiary education	40
Person affected by leprosy 4	Male	65	Tailoring	Primary education	9
Person affected by leprosy 5	Male	33	No occupation	Primary education	20
Person affected by leprosy 6	Male	41	Security guard	Primary education	20
Person affected by leprosy 7	Male	42	Tailoring	Primary education	20

**Table 2:** Participant information THs and CHWs working with people affected by leprosy

Participant	Gender	Age	Occupation	Educational level	Years experience working with people affected by leprosy
TH1	Male	55	Traditional healer	Primary education	12
TH2	Male	60	Traditional healer	No formal education	15
TH3	Male	42	Traditional healer in senior leadership position	Tertiary education	10
TH4	Male	46	Traditional healer	No formal education	13
TH5	Male	47	Traditional healer	No formal education	13
TH6	Male	43	Traditional healer	Secondary education	11
TH7	Male	62	Traditional healer	No formal education	12
TH8	Female	58	Traditional healer	No formal education	10
CHW1	Male	47	Community health worker	Secondary education	6
CHW2	Male	24	Community health worker	Tertiary education	4
CHW3	Male	33	Community health worker	Secondary education	8

### Study procedures, data collection and interview tool

Before the commencement of fieldwork, the topic guides for the semi-structured interviews for the different participant groups were developed using ethnographic theories, such as Kleinman’s Explanatory Models and the Health Care Systems Model (41, 42), and topic guides from similar studies. Recruitment of participants was facilitated by GLRA Sierra Leone through recruitment of the participants according to the eligibility criteria. In addition, GLRA supported courtesy visits to the local leaders of the participant groups and community chiefs. During the field visits, participants that matched the inclusion criteria were asked to be included in the study. The interviews were conducted as semi-structured interviews using the topic guides in May and June 2024 in the Western District of Sierra Leone. To avoid financial burden on the participants, and to accommodate the potential disabilities of people affected by leprosy, they were interviewed directly in the communities, either in their own homes, the community health clinics or their traditional health centers. All interviews took place in a secluded setting, ensuring the privacy and anonymity of the participants and were audio-recorded. Except for one interview of each participant group, all interviews were conducted in the local language Krio and translated by LHK from GLRA Sierra Leone. Three of the interviews were conducted in English without translation by AA from Maastricht University. To ensure accuracy of the translation and mutual understanding, medical or other unclear terms were explained by the translator. After conducting the interviews, the audio-recordings were transcribed verbatim using Microsoft Word. In the transcription process, the Krio translations of the questions and the direct answers of participants were not transcribed. All remarkable situations, and questions that arose during field visits, for example, to the leprosy hospital in Lakka or the leprosy colony, were discussed with the field supervisor from GLRA Sierra Leone to contextualize the findings from the interviews.

### Data management and analysis

The transcripts served as a basis for the content analysis. Atlas.ti (Version 24) was used for coding, which was conducted separately for the different participant groups, to keep the different perspectives separate from each other and minimize the loss of data through abstraction (43, 44). The analysis consisted of inductive coding, which generated codes, subthemes and themes (45, 46). Thematic content analysis was used as a method to report the key elements of the participant’s answers and categorize and compare data from various perspectives (47, 48). Orientated on the six-step-model for qualitative data analysis proposed by Braun and Clarke (47), potential themes were established from the codes. Next, the themes were revisited and integrated or connected if possible and reasonable. The codes and (sub)themes were added to the codebook in Microsoft Excel. After gathering all the codes from the interviews and revisiting and refining the codes and (sub-)themes, the final themes and subthemes were selected. They were then used to generate results and answer the research question and the respective sub-questions. This approach to analysis made it possible to maintain the richness and diversity of the data while also making the results more tangible.

### Ethics statement

Ethical Approval has been obtained from Maastricht University (*FHML/GH_2024.030)* and the Sierra Leone Ethics and Scientific Review Committee (*013/05/2024*) prior to data collection. Voluntary fully informed written consent was obtained from all participants prior to the interview, with emphasis on the right to withdraw at any point of time without giving a reason and without any repercussions. Moreover, permission to conduct research was granted by local community chiefs, the National Association for People Affected by Leprosy (NAPAL), the head of THs in Sierra Leone and the supervisor of the CHW program in Western District. The interviews were recorded and transcribed, while community and city names were substituted, to guarantee confidentiality of the data provided by the participants throughout the research and publication process.

## Results

**Fig. 1:**
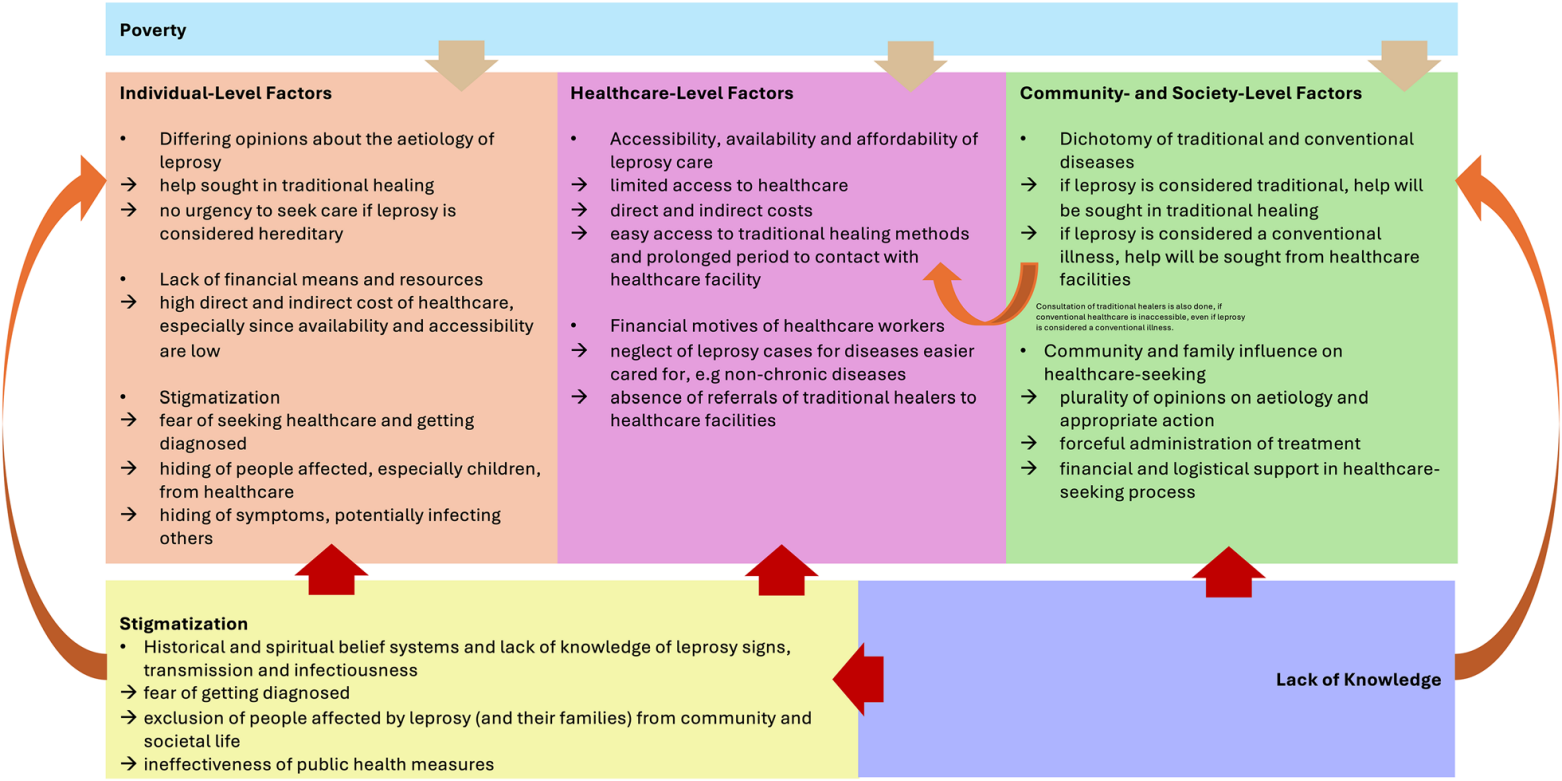
Influential factors on healthcare-seeking behaviors of people affected by leprosy in Sierra Leone

This qualitative exploratory study investigated the major influential factors on the healthcare-seeking behavior of people affected by leprosy in Sierra Leone. For a more holistic understanding, the results have been grouped according to a modified version of the Socio-Ecological Model proclaimed by McLeroy, Bibeau and Steckler (49) (Fig. 1).

### Individual level factors

*Perception of disease etiology* The illness experience and the respective interpretation of people affected by leprosy can hardly be generalized. However, the perceived etiology of their disease is a major influential factor on the healthcare-seeking behavior of people affected. Among the participant groups of this study, there were different opinions about the characteristics of leprosy. It was generally associated with disfigurement and disability. Moreover, it is seen as a cause for poverty of people affected by it. Three different etiologies of leprosy were distinguishable from the interviews and seem to circulate within society. The first theory about the cause of leprosy was that it is an inherited disease. Many of the participants considered leprosy to be family related or hereditary, given from generation to generation. This also often results in blame of family members that were affected by leprosy:

*“In my opinion, it is something hereditary, because my uncle from my mother’s side, he also had leprosy. So, I think I might have gotten through him, that’s what I think.” (Person affected by leprosy 6)*

The second perceived cause of leprosy was of spiritual etiology. Participants reported that leprosy is caused by black magic, bewitchment with evil spirits or by God. This happens either randomly or as retribution for engagement in immoral activities. Remarkably, the theories of hereditary and spiritual causality also appear intertwined, with bewitchments given from one family member to others:

*“And leprosy can also be acquired by a curse, someone may have been cursed by the communities or they have been engaged in some bad activities, so it’s sometimes caused by Karma.” (CHW2)*

Third, leprosy was perceived to be caused by bacteria or germs, and the lack of bad hygiene and sanitation:

*“There are times, because leprosy is an infectious disease, which normally caused by mycobacteria called Leprae. From contaminated water and dirty places, there we get the bacteria. So, it can be caused by bad hygiene, bad sanitation. That can all cause leprosy.” (CHW1)*

Leprosy is often considered infectious and transferrable, with the transmissibility ranging from low to highly contagious. These characteristics were found to coexist with the abovementioned etiologies, meaning that for instance hereditarily acquired leprosy is also transferrable:

*“I also do believe that it’s contaminated [contagious], so you don’t use the same drinking cup as someone who presents with leprosy. It can also transfer through sharing cups or spoons. You don’t exchange cutlery with them.” (TH2)*

In essence, there was no pattern on participant groups and related cause. Many different theories are circulating within the communities and the participant groups, subject to change through contact with each other, for example, leprosy as an inherited curse that can be given to the children of people affected by leprosy. This may also be the reason why there is no clear idea about whether leprosy is curable through traditional healing or if it needs biomedical treatment. The perceived etiology of the leprosy seemed to influence the urgency in which the treatment needs to be sought. If leprosy was believed to be hereditary, there was no need to seek prompt medical assistance:

*“So, my family, we are thinking is hereditary, it’s a family thing. So, there were less efforts to take me for my treatment with conventional medicine to the hospital.” (Person affected by leprosy 1)*

A common reason for delay in diagnosis and treatment at healthcare facilities was the attribution of leprosy to a spiritual cause, for which help was sought from the THs instead of biomedical healthcare. The perceived etiology of leprosy influences the healthcare-seeking behavior directly, as it results in seeking either traditional or biomedical healthcare, in accordance with the societal dichotomy of diseases. Attributing leprosy to supernatural or hereditary causes can render seeking immediate healthcare seem unwarranted and a poor use of resources. Different opinions on the transmission, symptoms and etiology of leprosy thus contribute significantly to a delay in diagnosis and seeking healthcare in other directions that will not contribute to diagnosis.

*Financial means and resources* Due to limited availability, accessibility and affordability of healthcare services, financial means and resources of individuals and families determine the care they can attend. People affected reported difficulties in managing their treatment-seeking efforts due to financial constraints:

*“Where we live, it is a remote area. I had some other treatment, but we didn’t know it was leprosy. And poverty is another issue, that led to my condition getting worse, we couldn’t move away from the village to seek help somewhere.” (Person affected by leprosy 5)*

Meanwhile, remote location of healthcare facilities, and resulting challenges in accessibility, poses another obstacle to prompt diagnosis and treatment. Long journeys inevitably result in loss of income, since time is allocated toward healthcare-seeking rather than production of income:

*“So sometimes getting into a health facility takes around 2-3km [distance to the next (community) health center], so those people can’t go. For the next hospital it was around 18-20km walking.” (Person affected by leprosy 1)*

The results highlighted that even if people affected decide to seek healthcare, they are often hindered by the lack of financial and time resources to do so. Their families, therefore, play a vital role in supporting the individuals affected with financial and logistic aid.

*Fear of stigmatization* Stigmatization and discrimination of people affected by leprosy seem to influence the healthcare-seeking behavior of persons affected by leprosy on individual, healthcare and societal levels. Stigmatization is present in society, communities and families of those affected by leprosy. During the interviews, persons affected by leprosys reported the frequent experience of exclusion from society and their own communities. It seems to be linked to common ideas associated with leprosy etiology, such as bewitchment by evil spirits, or linked to the perceived risk of infection when interacting with a person affected by leprosy:

*“So, I started experiencing some stigma, people didn’t want to eat with me or come close to me, some even requested to my mom that they shouldn’t let me live at home. So, I was living in the bush around the house where we lived.” (Person affected by leprosy 5)*

Stigmatization and discrimination were reported to be common within families affected by leprosy. Individuals are excluded from their families, not being allowed to participate in family life or even to live with their families:

*“The people, some have been abandoned, in the evil forest, because of their conditions. Some of them, they died in the evil forest, because they don’t allow them to come back to live in the community.” (TH3)*

Moreover, since in some cases, leprosy is believed to be hereditary, sometimes whole families of persons affected by leprosy are excluded from the community. To avoid stigmatization as a family, persons affected by leprosy—especially children—are hidden from the communities. Moreover, family image in the community seems to take precedent over the adequate care for the individual affected by leprosy in some cases. To keep the good family image, children affected by leprosy are hidden from the public—and, therefore, adequate care:

*“When our young girls, young women, got leprosy, first of all, stigmatization is common. They will keep them in the communities, they don’t give them to us. They’re not allowed marriages; they don’t take public offices.‬” (TH3)*

As observable from the quotes, stigmatization and discrimination frequently cause mental health problems. In addition, self-stigmatization has been mentioned by some participants. Most of the respondents claimed, that mental health issues could be averted through proper diagnosis, treatment and laboratory confirmation of the treatment to reassure cure of the disease. However, they acknowledge that alone would be insufficient, as the exclusion from families and communities also take a toll on their mental health:

*“You’ll isolate yourself before people will isolate you. You’ll say, because of my condition, I will not go closer. I will be afraid and ashamed to go closer, for people not to say: ‘what is smelling?’ So, such question you will not be able to answer, so best is straight to leave.” (Person affected by leprosy 3)*

*“If there is a laboratory confirmation, that there is still leprosy or not, that could make the person courageous, that could make them mentally well and feel better.” (Person affected by leprosy 6)*

As form of social support and coping mechanism, people affected often live together in leprosy colonies, where people who were affected by leprosy and subsequently excluded from society live together to support each other:

*“Life is not easy for leprosy patients. There is shame on us. We are always at the back of the queue. No one values us. Society doesn’t recognize us. It’s because we are in the same colony that we are coming together and interacting with each other, but it’s very difficult for us. I normally feel ashamed to interact with people because of my leprosy.” (Person affected by leprosy 5)*

THs and CHWs experience reluctance to seek treatment by people affected by leprosy, since they are afraid of being stigmatized once they are diagnosed with leprosy:

*“We do not force them [to get diagnosed at a health center], because some become very reluctant. They will say that they’re afraid to fall into stigmatization.” (TH3)*

All the CHWs highlighted the importance of counseling and mental health care for people affected by leprosy. They stated that attaining to the mental health and well-being of the people affected by leprosy within their designated community was one of their responsibilities. Trust seems to play a key role for CHWs to be able to encourage people affected to seek adequate treatment and for treatment adherence. Counseling and mentorship were emphasized as important factors in trust-building and for dismantling (self-)stigma and the discrimination in society:

*“I would also have to talk to them for them to realize that it’s dangerous but it’s not the end of someone’s life, and it’s curable. And I can interact. It should not lead to self-stigma. Some kind of mentorship.” (CHW3)*

Hesitancy to get diagnosed and treated, and family and community members hindering people affected by leprosys from getting diagnosed, result in diagnostic and treatment delays. This additionally perpetuates the stigmatization and discrimination, since the possibility of developing deformities and disabilities increase, as does the risk of spreading the disease. However, CHWs and other trusted community members seem to be able to influence the healthcare-seeking in a positive way.

### Healthcare system factors

*Availability, accessibility, and affordability of healthcare options* Even though there is a dichotomy of diseases and appropriate healthcare-seeking action, it appears that this is often trumped by practicalities, such as availability, accessibility and affordability of the healthcare options within the communities. THs are a common first point of contact to seek medical attention, and usually responsible for illnesses with supernatural and spiritual etiology. People affected by leprosy described several reasons for seeking traditional healing. In addition to beliefs in spiritual or supernatural causes of leprosy, the lack of knowledge about alternative options was a reason to seek traditional healing. Most of the PALs stated, that THs were the first point of contact they sought for help for their illness episode, due to their accessibility and availability within the communities:

*“Traditional healers, they’re easy because they are around in the community.” (Person affected by leprosy 1)*

Other factors that led to preference of traditional healing methods over biomedical treatment were frustration with biomedical treatment and mistrust in biomedical healthcare. For example, THs were frequently consulted, when the patient already had been to the hospital for a certain amount of time, maybe even suspecting a spiritual cause of their illness:

*“For example, if they have been in the hospital for a long time and they realize that they have been shot with a black magic gun, which the conventional medicine cannot treat, they will come to us.” (TH8)*

However, there were also cases, where people sought help from the CHWs first. Their prompt availability of CHWs and their affiliation with the health center were the main motivations to seek help from them. In addition, the free examination and referral services were essential factors that led people affected by leprosy to report to CHWs:

*“As a CHW we are based in the community, so if there is something weird detected, we’re the first point of contact to capture it and refer it to the hospital. There are also times we do outreaches to the communities, outreach sessions.” (CHW1)*

CHWs, who are often recruited directly from their communities, enjoy a high level of trust among them. However, they acknowledged that there remains a lack of awareness about the CHW system within the broader community:

*“They are quite confident that I am a son of the soil. I am their brother, I am their son, I am their everything. I am part of the community here, so they’re quite confident in me.” (CHW3)*

Lack of community knowledge of alternatives to traditional healing, like the CHW program, and local disease concepts ultimately resulted in the consultation of THs:

*“They often present to the traditional healers, because they have no idea that there is someone they should report to.” (CHW3)*

The remote location of healthcare facilities and, therefore, challenges in accessibility and availability were described. This lack of availability and accessibility for some of the people affected resulted in lack of compliance and treatment adherence:

*“So I went to a general hospital, it was quite a long distance from my village. They were also experiencing drug stockouts, so there was continuous interruption of my treatment, and I had to stop again.” (Person affected by leprosy 6)*

People affected by leprosy reported difficulties in managing their treatment-seeking efforts due to financial constraints. Traditional healing was perceived to be more affordable than biomedical treatment and was, therefore, preferred:

*“Where we live, it is a remote area. I had some other treatment, but we didn’t know it was leprosy. And poverty is another issue, that led to my condition getting worse, we couldn’t move away from the village to seek help somewhere.” (Person affected by leprosy 5)*

*“Now, it’s cheaper to go to traditional healer than the medical facility treatment, it’s quite expensive.” (Person affected by leprosy 1)*

The wide availability and easy accessibility of traditional healing within the communities, and the lack of biomedical healthcare options or the knowledge thereof, commonly makes the THs the first point of contact in the communities. Availability within the community and ease and speed of access were named as key factors to prefer traditional treatment over biomedical treatment, even when the perceived etiology of leprosy was not spiritual. Lack of affordability and financial constraints of people affected by leprosy seem to play a role in healthcare-seeking, and their adherence to treatment.

*Financial motives of healthcare practitioners* Even if people affected by leprosy seek healthcare promptly, the financial motives of healthcare providers often pose another obstacle for a swift diagnosis and administration of adequate treatment. The frequent absence of referrals from THs to biomedical medicine was a recurring concern, often attributed to economic motivations rather than a commitment to patient care. Nevertheless, people affected reported that health workers acted with a profit-driven motive as well, where leprosy care was neglected in favor of more lucrative diseases and diseases easier to care for:

*“He decided for the fast way to get money instead of doing the work with Leprosy, that they employed him for. […] He had the knowledge, but he was only interested in getting money at the time and not Leprosy. So, when I went there, he took a long time for treating other’s illnesses [Person affected by leprosy 1 mentioned Malaria earlier in the interview]. I was sitting there and sitting there, me and my sister, just waiting for him. He really did not care about Leprosy, but he was employed for Leprosy. So, you had a long waiting time. We even give him money, you see, my sister, for him to take care of me.” (Person affected by leprosy 1)*

THs frequently described their role within the healthcare system as first point of contact, responsible for illnesses with supernatural and spiritual etiology. They report that their livelihood is heavily reliant on good reputation within the community and recommendations of former patients. Economic motives seem to play a role in their endeavors, as it was mentioned by people affected, as well as CHWs. Amongst the THs themselves there was no consensus about the economic motive. Some of them admitted to take money from patients, while others claimed to take no payment and rely on prayers and material gifts to get paid:

*“They never tell you to go to the hospital. Because those people also need the money, you know the situation of the country. When someone go there he know this person have money, so they just get money from you. So, they didn’t tell you go to the hospital where we have leprosy people, no one ever do that.” (Person affected by leprosy 1)*

*“Traditional healers are money-makers; they don’t care about the health.” (CHW2)*

The opinions about biomedical medication differed amongst the THs. On one hand, some reported that leprosy medication is free of cost at the health centers and hospitals. On the other hand, those who claimed to be able to cure leprosy stated, that medication at the hospital is more expensive than traditional one, and subject to availability:

*“One of the two things people […] say when you go to the English medication, the conventional, you pay more money, huge amounts of money. That is one. Two, they would not be able to get access to medication. But in the traditional medication is very low, the cost is very low then your access to medication is very fast.” (TH3)*

Most people affected described feelings of condemnation, resentment and anger toward the traditional treatment and the healers. Some people affected by leprosy experienced that THs guess the condition and randomly assign the treatment, and that they would concentrate on treating symptoms rather than the underlying disease. Moreover, people affected by leprosy criticize the financial motives of THs. Treating patients to earn money seems to take precedence over the care for the patient’s health:

*“But I wasn’t happy with the traditional healer’s treatment, and I really condemn that, and I keep advising my colleagues that are newly diagnosed that they should not go to the traditional healer for treatment.” (Person affected by leprosy 2)*

*“Even today they know about leprosy, but because they need money, they just take care of the case, even they don’t know what to do.” (Person affected by leprosy 1)*

Interestingly, some of the THs described to be part of a referral system between THs and the biomedical health sector. These THs reported to refer people affected by leprosy if symptoms persisted, or they got words. The first option is to refer the case to another traditional healer. This, however, bears the risk of reputational damage, and consequently loss of clients:

*“Sometimes, when you refer people to some other traditional healers, you get quite some bad feedback […], so once they don’t get the treatment from the person you refer them to, you will get the blame. That is why sometimes, we don’t do referrals, we prefer referring to the hospital, instead of a colleague.”‬ (TH1)*

The second option involves referring patients to a health center or hospital. Some traditional healers described their role within a referral system, with responsibilities such as facilitating re-integration of people affected in their communities. This includes providing counseling, following up with individuals after the hospital, and ensuring adherence to their treatment regimen. This is contrasted by the description of financial motives of traditional healers, which seem to exist alongside this motivation for referrals.

### Societal and community-level factors

*Choice and evaluation of the adequate healthcare option* To understand healthcare-seeking behavior and the pathways of people affected by leprosy within the healthcare sector, the general concept of choosing and evaluating healthcare alternatives present in the communities must be understood. Participants described that there are two different types of illnesses existing, which require different treatment and management approaches. While some diseases were ideally treated by medical doctors, other diseases were best cared for by the THs:

“*I don’t think all kinds of diseases should be treated by the traditional healers; some diseases require conventional treatment.” (CHW2)*

On one hand, some diseases of biomedical origin could be best treated by biomedical treatment. In this case, this entailed treatment at the hospital and health centers by nurses, doctors or other clinical personnel. On the other hand, diseases with a mystical or spiritual etiology were best presented to THs for cure. Illnesses, that were believed to be caused by supernatural phenomena, such as witchcraft, spirit possession and black magic, should be handled by the TH. Moreover, it appears that it is also deemed appropriate to present cases of psychiatric illnesses to a TH:

*“But the mental illness or their psychiatric problems, use of devil, witchcraft, those have to be addressed by traditional healers, even if you lie down for years.” (TH3)*

There appears to be a dual approach to classifying diseases, either as of biomedical or spiritual and mystical etiology. Accordingly, the adequate healthcare action is to either seek help from a TH, or from biomedicine specialists.

*Community and family influence on the healthcare-seeking* Individuals affected by leprosy reported different approaches to navigating their healthcare-seeking and treatment. Although most of them managed and organized their care independently, family, friends, neighbors, and community members seem to influence their healthcare decisions. This was the case for both the primary healthcare-seeking and for re-evaluation of treatment options after unsuccessful treatment. Communities and families assist in the decision-making process through providing knowledge about healthcare options. However, participants described a lack of knowledge on leprosy in the communities, causing many different ideas about etiology to circulate. This plurality of opinions and options was described as a hindering factor to prompt and adequate treatment, as they encountered difficulties in identifying the appropriate healthcare option. Moreover, they explained that there was a lack of awareness of leprosy services within the communities, leading to consultation of THs:

*“There was lack of awareness about leprosy and people didn’t know about the condition of the disease and what the cause was and how it’s treated.” (Person affected by leprosy 1)*

To counter this, participants expressed the need for more community education on leprosy and enhanced community involvement in leprosy care:

*“There should be involvement of community chiefs, pastors, teachers, other religious leaders. We need to involve the community as well, to raise awareness, the community is key in that sense. I think community awareness should be at the forefront.” (CHW3)*

In addition, some respondents expressed, that they had no say and autonomy in their decision-making regarding treatment options, as they were patronized by family and community members. This led to forceful administration of traditional treatment, since family or community members assumed a spiritual cause. Moreover, refusing traditional treatment was believed to indicate that one might be responsible for their disease, further impeding free decision-making. In addition to that, participants described the premature termination of biomedical treatment, due to high costs for mobility and time-consuming travels to the healthcare facility:

*“So, my family said, ‘you’ve healed, there is no need for you to go again’. So, at that time I was just a child, whatever my parents say, I will exactly listen to.” (Person affected by leprosy 3)*

*“That’s why people often go there with their medical condition. Sometimes the decision is also taken by their families, and they have no say in their health decisions.” (CHW3)*

However, in many cases, other community members supported the healthcare-seeking. This support was mainly of financial nature but also included material and logistical support. For families with children affected by leprosy, the parents oversee coordination and organization of diagnosis and treatment. Mothers typically took charge of coordinating treatment seeking, while fathers often served as the ultimate decision-makers:

*“Well, it was those who were aware of the disease [from the community], they took me to places to seek for treatment. They were the ones who pushed me to go. My uncle from my mum’s side, he had also some idea of leprosy, he also knew that leprosy couldn’t be cured by a traditional healer.” (Person affected by leprosy 7)*

*“No other family members get involved, it’s the problem of the dad and mom, to take care of their kid, no one cares.” (Person affected by leprosy 1)*

*“So, my dad was reluctant. So, I wasn’t allowed to come. And it kept on going and going and going.” (Person affected by leprosy 1)*

Families and communities appear to play an essential role in the evaluation of treatment options for their ill family members, mostly through providing financial support and knowledge on treatment options. Nonetheless, the involvement of families is associated with a loss of autonomy in the decision-making process for people affected by leprosy. It can also lead to delays in treatment due to differing opinions among family and community members on the appropriate course of action.

*Stigmatization at family- and community-level* People affected by leprosy often experience family rejection and public discrimination due to a variety of factors, such as misconception about its transmission, association with bewitchment, and the fear of infection. Disabilities and deformities contribute to the stigmatization of individuals affected by leprosy. Visible deformities, that can remain after successful treatment, can reveal the diagnosis and are frequently seen as indicators of infectiousness. This caused discrimination in the form of exclusion from job interviews or viewings for housing, hindering re-integration into society:

*“Even though you have been cured, you still have deformities, people will say the sickness started again. They will not drink from the same cup, lie in the same bed, or eat together. So that stigmatization is still ongoing.” (Person affected by leprosy 3)*

Additional difficulties arose from disabilities that people acquired through leprosy. For example, they were unable to continue working in the jobs they were trained for. Amongst the people affected by leprosy, many noted that though living with disabilities, they were perceived of not being useful for society anymore:

*“First of all, this is a disease that is debilitating, you will be marginalized, everyone would be running away from you. No one would get close to you, everyone will shy away from you, no one would come to where you live, no one would want to live with you anymore. […] The stigma it carries is so much, even with complete care, it is not easy. It has a serious mental health toll. Because people feel you’re not useful for society anymore, so you get totally neglected.” (Person affected by leprosy 4)*

Participants blamed the lack of knowledge in society and the spread of misinformation about leprosy for perpetuating their stigmatization and discrimination:

*“They were the ones who started misinforming the public about the nature of the disease which they have no knowledge about. […] The sisters were the cause for the neglect of my family. Because they had no idea, and they took things out of proportion.” (Person affected by leprosy 1)*

## Discussion

The findings from this study demonstrate, that healthcare-seeking behavior is influenced by a multitude of different factors on different levels of the healthcare-seeking process. The limited accessibility, affordability and availability of leprosy care, perceived etiology of leprosy and the stigmatization of people affected by leprosy were the main influential factors on diagnosis, treatment and management of leprosy. The role that THs and CHWs play in leprosy care is significantly influenced by the omnipresent poverty in the society and related financial motives. Communities and families seem to influence the healthcare-seeking depending on their understanding of leprosy and their resources.

### Dichotomy of diseases

In general, two different types of illnesses are distinguished in the society in Sierra Leone, which both require different medical attention. Diseases of biomedical etiology need to be seen by personnel from the biomedical health sector. Illnesses with a traditional or spiritual etiology are best attained by a TH. Despite this clear distinction, healthcare-seeking is ultimately guided by other factors as well, such as availability, accessibility and affordability of healthcare choices and stigmatization. THs are widely available and a common first point of contact for those who fall ill. Often, help is sought there, even when the underlying cause is neither spiritual nor mystical. Their livelihood depends on community trust and reputation, which in turn might lead them to prioritize financial stability over patient referrals. Resultingly, THs are attributed financial motives for their work as healers, and they stand in concurrence to the biomedical health sector. Some participant reported that referral to other THs or the biomedical healthcare system is only organized when THs deem their patients unsuitable for traditional healing, or their symptoms worsen. Besides traditional healing, biomedically trained CHWs attain to the health of the community they serve in. They enjoy high levels of trust from their community and are strongly associated with the healthcare system. Despite the existence of the CHW program, there is often a lack of accessible, available and affordable alternatives to traditional healing within the communities, hence traditional healing is sought. Three main theories are circulating within the communities about the etiology of leprosy: hereditary origin, spiritual cause and caused by bacteria or germs. The coexistence of these theories may also be the reason why there is no clear understanding about whether leprosy is curable through traditional healing or needs biomedical treatment. Remarkably, across different participant groups, the signs and symptoms of leprosy were appropriately associated with skin lesions—independent from the perceived origin. This can serve as a contributory factor in early detection and healthcare-seeking behaviors and underscores the need for health promotion efforts to not only clarify the causes of leprosy but, more importantly, to emphasize its signs and symptoms, urging individuals to take them seriously and seek timely medical attention. Thus, the perception of the etiology can be eliminated as a potential hindering factor in seeking timely care.

### Financial means and resources, financial motives and poverty in society

This study demonstrates, how the overshadowing poverty in Sierra Leone influences the healthcare-seeking and decision-making of individuals within the healthcare system. According to the World Bank, 43% of the population of Sierra Leone are living below the international poverty line of 1.90$ per day, and 93% are at risk of impoverishing health expenditures (50, 51). The status of the National Health Care Services, as well as the access to it is poor (30).

In the context of poverty, it is unsurprising that practical considerations, particularly financial constraints, often take precedence over the belief system of different etiologies of diseases. Moreover, the time-intensive nature of seeking treatment also plays a significant role. Ultimately, the time dedicated by affected individuals and their families to pursue treatment is time that could otherwise be spent working and earning an income. This is reflected within people’s actions, such as the premature termination of biomedical treatment or the turning from the hospital to a TH in cases of chronic diseases. People tend to be economically cautious, carefully managing their limited resources, financial and timewise. This interlinks with the lack of accessibility, availability and affordability of healthcare services. If they are neither accessible and available, nor affordable, additional financial and time sacrifices will have to be made to seek treatment. In the communities the gap is closed by high accessibility of traditional healing practices. Thus, healers are commonly consulted, even if etiology is not perceived to be fit for traditional healing. People affected by leprosy also include community perspectives into their decision-making. Biomedical care is often perceived to be expensive; therefore, THs are consulted. THs also contribute to the perceived high cost of biomedical healthcare, thereby encouraging a steady inflow of clients to them. These findings align with studies conducted in Sierra Leone for child health and Ebola, stating that local beliefs and the availability of healthcare services are major influential factors in healthcare-seeking (24, 25).

### Family and community influence

Due to the costs and poor availability of leprosy care, family and community support is often vital. They provide financial aid, logistical support, and influence healthcare decisions. Although many patients take responsibility for managing their own healthcare, the involvement of family members and the causes attributed to leprosy often complicate their efforts. Not only families, but also communities provide these forms of support for healthcare-seeking endeavors of their community members. This confirms the findings of prior research on the importance of social networks in healthcare-seeking in Sierra Leone (37). When healthcare efforts seem to fail, the exploration of alternative options is frequently guided by the input of both the community and the family.

The lack of awareness of healthcare services within the community, such as CHWs, hampers prompt diagnosis and treatment seeking. Participants of the study expressed the need for increasing community awareness of leprosy and healthcare services, and the engagement of community members in education on leprosy. Trainings of community leaders, THs and health workers on leprosy has already been conducted in the past and shown promise in improving detection rates and correcting misconceptions (30). Studies from Indonesia and Nigeria confirmed the beneficial effects of community education on case detection and attitude toward people affected by leprosy (52, 53). The community reliance on traditional healing can be used through education of THs to detect and refer leprosy cases. Subsequently, the people affected by leprosy assume spiritual cases, will not be overlooked. To further increase the availability and accessibility of leprosy care, CHWs in this study underscored the need for more training and empowerment, to successfully contribute to the provision of healthcare in the communities. This has already been shown to be a successful measure to improve timely diagnosis and referral in studies from Ethiopia and Nepal (54, 55). Meanwhile, studies conducted in Sierra Leone concluded, that the involvement of primary healthcare workers is essential for elimination of skin NTDs (34, 35). In general, the delivery of specialized care in low-resource settings like Sierra Leone is impeded by shortages in trained personnel, making innovative approaches necessary to increase accessibility of care for people affected by leprosy (56).

As the findings indicate, reputation and personal recommendations are highly valued within communities and strongly influence healthcare-seeking behavior. However, many participants expressed skepticism toward the formal healthcare system. THs, who are often trusted members of the communities and deliver alternative explanatory models of diseases to the biomedical causes, remain a preferred option for many. CHWs play a crucial role in bridging this gap by acting as trusted mediators between communities, where they are recruited from, and the healthcare system. By delivering culturally sensitive and patient-centered care, they can help reshape perceptions of biomedical healthcare, making it a more trusted and accessible option. As trust grows, more individuals may turn to professional healthcare services, leading to earlier diagnosis and timely treatment of leprosy, ultimately improving health outcomes.

### Stigmatization

Besides poverty and financial constraints, stigmatization also influences the decision-making of people affected by leprosy. Leprosy is widely known, and so are the negative attitudes and stereotypes that are associated with it. This often implies severe repercussions on the social status of the family, friendship, potential partners and work. People not only fear the visible disfigurement of face, hands and feet that leprosy may cause, but also the social implications of stigmatization and discrimination (57). Before there was treatment for leprosy, the exclusion of people affected may have been a protective public health measure; however, it inspired today’s belief that people affected should be isolated from society (57). Similar perspectives on stigmatization as a barrier to leprosy care have been found in studies conducted in Nigeria and Cameroon (9, 33). The fear of stigmatization creates an unfavorable environment for early case detection and impedes leprosy control efforts, for instance, families reject the presence of healthcare workers who do contact screening as it exposes the family’s health status. Thus, delayed reporting increases the risk of transmitting the infection to others. Stigmatization itself impedes mental health, socioeconomic status, and the overall quality of life. Leprosy in Sierra Leone is commonly associated with disability and disfigurement and seen as a cause of poverty. Moreover, leprosy is known to be infectious. Most people, however, are under the impression, that disability and disfigurement, which often remain after successful treatment, are signs of active and transmissible leprosy. People affected by leprosy commonly experience stigmatization and discrimination based on their condition. This entails isolation from their families and communities, difficulties in re-integration after successful treatment and neglect by society. The fear of stigmatization and the subsequent delay in healthcare-seeking lead to a vicious cycle. Delayed treatment exacerbates the condition and, therefore, reinforces stigmatization, further discouraging healthcare-seeking. Most often, there is no adequate mental health care provided by the different sectors of the healthcare system. Lack of awareness fuels stigmatization on community and societal level, leading to exclusion from education and social life. Consequently, this social marginalization affects individual well-being and mental health and reduces economic productivity in Sierra Leone. Community education can reduce stigma in two key ways: early detection prevents deformities that fuel discrimination (15), and increased knowledge about leprosy’s curability and low transmissibility reduces fear. Through increasing of community awareness, community cooperation and resources are more likely to be mobilized for healthcare-seeking efforts (58). However, as healthcare-seeking behavior is guided by different factors, fighting stigmatization is not the solemn solution for improving early diagnosis and treatment of leprosy.

### The ultimate choice of healthcare options and influential factors

Despite an apparent belief system, where spiritual and mystical, and biomedical etiologies of leprosy and other diseases are differentiated, and call for an adequate healthcare option, the reality often looks different. People affected by leprosy seem not to adhere to the dichotomy of diseases singularly, but adjust their healthcare seeking according to economic resources, availability and the chronic persistence of diseases. Thus, the perceived origin does not determine the treatment choice. This strongly aligns with studies conducted on Buruli ulcer, another chronic skin NTD, conducted in Cameroon (20). The findings suggest that factors beyond belief systems, such as treatment availability, trust and mistrust in healthcare, cost, and stigmatization, play a decisive role in guiding treatment options. Economic reasoning was also found to take precedence over the belief system in place, which shaped not only patients but also the healthcare-seeking of families and communities. Consultation of traditional healing can also be seen to avoid the often debilitating cost of biomedical healthcare. Mistrust in biomedical healthcare and the fear of economic exhaustion has also been shown to be a factor of diagnostic delays in other studies (59, 60). The attribution of diagnostic delays to belief systems is a simplification of the real-world situation and places the responsibility on people affected by leprosy, neglecting the structural issues within the healthcare system. More available biomedical care and more trust in healthcare can foster the usage of healthcare services. If aligned with peoples’ careful economic considerations, healthcare systems can serve as a more frequent choice.

### Limitations and strengths of the study

The use of semi-structured interviews generated rich data for addressing the research question. While triangulation of the results with focus group discussions could have enhanced validity, the consistency of participants’ responses and alignment with previous research on leprosy healthcare-seeking and healthcare-seeking in Sierra Leone support the reliability of findings.

To avoid bias through translation, some of the interviews have been conducted in English directly. The findings from the translated interviews proved to be consistent with the ones that were conducted in English directly, showing that the translation did not severely alter the understanding of the information disclosed by the participants. A measure to further enhance reliability was the data triangulation during the analysis process. In addition, through discussing and contextualizing important insights from the interviews during discussions with the field supervisor and field visits, understanding of the results was improved.

Despite sincere efforts to include women affected by leprosy, none of them matched the inclusion criteria, as none of them had consulted a TH in their healthcare-seeking efforts. Morrison (58) notes, that growing knowledge on healthcare availability amongst women, and a more universal belief that leprosy is curable, might contribute to the immediate presentation to the professional health sector. Contrary, Okoro et al. (61) describe intersectional invisibility experiences of African women in healthcare encounters, which may contribute to the reluctance to seek care. Whether this absence in the study was coincidental, or indicative of a broader issue remains unclear. Further research is needed to study the influence of gender on the status of women, their healthcare-seeking the particularities of the intersection of gender and leprosy in Sierra Leone.

While the sample size limitation in this explorative research, particularly when divided into groups, may raise concerns about the robustness of the results, it is essential to highlight the valuable insights this study offers through exploring multiple perspectives of the different actors in leprosy diagnosis, treatment and management. As family and community members of people affected by leprosy turned out to be valuable players in the healthcare-seeking process, inclusion in further research is needed to unravel the complexity of community influences on healthcare-seeking behavior.

A key strength of this study is its inclusion of diverse ethnicities, unlike previous studies focused on a single ethnicity. This approach better reflects the realities of affected communities. In addition, by exploring multiple perspectives in leprosy diagnosis, treatment, and management, this research provides valuable insights into the cultural and social dimensions of the disease. It lays a foundation for more inclusive, participatory research in the future, guided by individuals with lived experience. Further studies should examine the influence of socio-demographic factors, such as education or quantifiable distance from healthcare facilities.

## Recommendations and conclusion

The effectiveness of interventions involving THs and CHWs should be systematically assessed, as they represent exemplary models of community engagement in facilitating the early detection of leprosy. In addition, the insights gained from this study regarding factors contributing to diagnostic delays should be integrated into active case-finding strategies and awareness campaigns within leprosy control initiatives in Sierra Leone. These findings are also critical for quality control and program evaluation. Moreover, an assessment of healthcare-seeking timelines is necessary to gain a deeper understanding of individual decision-making processes. Given the highly relevant findings of this study, ongoing leprosy control programs in the country should be critically evaluated, particularly in relation to active case detection methods and post-exposure prophylaxis, to optimize their feasibility and acceptability.

This research shows that delays in diagnosis and treatment of leprosy are caused on different levels of healthcare-seeking. Limited availability and accessibility of biomedical healthcare, coupled with financial constraints, hinder treatment-seeking. Even after initiation of treatment, high costs and time-consuming visits frequently lead to premature treatment termination, delaying recovery. Spiritual beliefs attributing leprosy to supernatural causes further obstruct timely care, especially when family members influence healthcare decisions through financial support and opinions. When symptoms persist into a chronic state, reliance on traditional healers increases. In addition, fear of a leprosy diagnosis and subsequent stigma deters individuals from seeking treatment.

These findings align with previous studies, highlighting that leprosy and diagnostic delays are not merely local or regional issues, not just specific to Sierra Leone. Diagnostic delays should be addresses in global and national zero leprosy roadmaps. The strong ties between communities, THs and CHWs present valuable opportunities for early diagnosis and treatment. Training of THs and CHWs, as well as efforts by the government can aid in addressing the lack of accessibility and availability of healthcare services. Community education can help in reducing stigmatization, and avoid the attribution of leprosy to spiritual causes, which results in seeking traditional healing. It is only through collective and collaborative efforts amongst THs, CHWs, communities and the government, that leprosy care can be effectively improved, and the barriers to healthcare seeking mitigated. More quantitative research is needed to determine the significance of education, economic capabilities and other factors on healthcare seeking. Furthermore, additional qualitative research is needed to stratify the complementary healing landscape, and to uncover the shaping of local disease conceptualizations. In addition, research focusing on the intersectionality of womanhood and leprosy is needed to understand the influence of gender on healthcare-seeking behavior.

## Data Availability

All important data are shared as anonymized quotes in this paper. Additional data required can be requested through the corresponding author with reasonable justification.

## Abbreviations

CHW(s): Community health worker(s)
TH(s): Traditional healer(s)
WHO: World health organization
